# Consequences of Maternal Vitamin D Deficiency on Newborn Health

**DOI:** 10.3390/life14060714

**Published:** 2024-05-31

**Authors:** Ramona Elena Dragomir, Daniela Oana Toader, Daniela Elena Gheoca Mutu, Iulian Alexandru Dogaru, Laura Răducu, Laurențiu Cezar Tomescu, Lavinia Cristina Moleriu, Anca Bordianu, Ion Petre, Ruxandra Stănculescu

**Affiliations:** 1 Doctoral School, “Carol Davila” University of Medicine and Pharmacy, 020021 Bucharest, Romania; ramona.dragomir@drd.umfcd.ro (R.E.D.); iulian-alexandru.dogaru@drd.umfcd.ro (I.A.D.); ruxandra.v.stanculescu@gmail.com (R.S.); 2 Department of Obstetrics and Gynecology, “Carol Davila” University of Medicine and Pharmacy, 020021 Bucharest, Romania; oana.toader@umfcd.ro; 3 Discipline of Anatomy, Department 2—Morphological Sciences, “Carol Davila” University of Medicine and Pharmacy, 020021 Bucharest, Romania; 4 Discipline of Plastic Surgery, “Carol Davila” University of Medicine and Pharmacy, 020021 Bucharest, Romania; laura.raducu@umfcd.ro; 5 Department of Obstetrics and Gynecology, “Ovidius” University of Constanța, 900527 Constanța, Romania; tomescu.cezar.laurentiu@gmail.com; 6 Discipline of Medical Informatics and Biostatistics, Department 3—Functional Sciences, “Victor Babeș” University of Medicine and Pharmacy, 300041 Timisoara, Romania; moleriu.lavinia@umft.ro (L.C.M.); petre.ion@umft.ro (I.P.)

**Keywords:** vitamin D in pregnancy, vitamin D deficiency, newborn outcome

## Abstract

Background and Objectives: Maternal–fetal gestational pathology is one of the biggest challenges in the field of health at this moment. The current study is designed to determine the effects of vitamin D on pregnancy, starting with the idea that impairment of vitamin D status is thought to be correlated with impairment of the newborn’s health. Materials and Methods: In this retrospective study, we tried to establish the link between vitamin D deficiency and maternal characteristics and also how it impacted the clinical status of the newborn. We analyzed a group of 260 patients: 130 pregnant women and 130 newborns, in whom vitamin D status was detected using the serum levels of 25-hydroxyvitamin D (25-(OH)D). Results: The results showed that vitamin D deficiency has a high incidence among pregnant women, as was presented in many important international studies. Our study also showed a positive, direct correlation between the mother’s and newborn’s vitamin D status. Conclusions: Taking into consideration that vitamin D deficiency has been correlated with many complications, both in maternal and newborn health, a serum level determination of 25-(OH)D is necessary in the first trimester of pregnancy, and after that, adequate supplementation is necessary in order to prevent any negative effects.

## 1. Introduction

Over the past decade, vitamin D has garnered increased attention due to its association with various deficiencies and health benefits. Numerous studies highlight the role of vitamin D in the development of pathologies during pregnancy, noting that maternal vitamin D status directly influences that of the newborn [1,2,3,4].

The effects of vitamin D are mediated by the vitamin D receptor, which binds the active form of vitamin D [1,25-(OH)2D], triggering both transcriptional and non-genomic responses. While vitamin D’s role in calcium absorption and bone metabolism is well-known, recent studies emphasize its newly discovered roles in various cell types, including modulating the innate and adaptive immune systems and regulating cell proliferation.

Adequate vitamin D intake is crucial during pregnancy for both maternal and fetal health. However, epidemiological data indicate that many pregnant women have suboptimal 25-hydroxyvitamin D (25-(OH)D) levels. Some studies have attempted to establish a link between low levels of 25-hydroxyvitamin D (the barometer of vitamin D status) and the occurrence of obstetric complications, but there is no consensus on this matter. Generally, pregnant women tend to develop vitamin D deficiency, reflected in both maternal and fetal serum levels of 25-hydroxyvitamin D, which should range from 30 to 100 ng/mL [5,6].

During pregnancy, the need for vitamin D increases, especially as the term of birth approaches. It is widely accepted that the physiological increase in the active metabolism of vitamin D, combined with the increased need for fetal calcium (particularly in the third trimester) and enhanced intestinal absorption, works to optimize vitamin D status during pregnancy [7,8,9,10]. 

Faustino R. Pérez-López et al. provided significant insights in a review on vitamin D supplementation during pregnancy, aimed at improving both maternal and fetal health. The review concluded that pregnant women need an additional 600 IU/day of vitamin D, and suggested that higher doses of vitamin D (1000–4000 IU/day) might yield better outcomes for both mother and newborn [11,12]. 

It is important to note that various lifestyle risk factors contribute to vitamin D deficiency in pregnant women. These factors include limited sun exposure, residence at higher latitudes, darker skin pigmentation, clothing practices, and inadequate intake of vitamin D. 

Several clinical studies have documented an association between low 25-(OH)D levels and adverse pregnancy outcomes such as pregnancy-induced hypertension, gestational diabetes, spontaneous abortion, preterm birth, and postpartum depression. These studies also highlight the impact of vitamin D deficiency on placental circulation dynamics [13,14,15,16]. Studies regarding maternal health have focused lately on the link between vitamin D deficiency and increased insulin resistance and the higher incidence of anemia due to vitamin D involvement in the regulation of erythropoiesis in the bone marrow. Also, results suggest that vitamin D deficiency can impact iron metabolism, potentially leading to anemia [17].

Many studies have tried to establish the link between vitamin D maternal status and newborn health. It is known that vitamin D is essential for fetal skeletal development and normal growth. Some studies suggest that vitamin D deficiency during pregnancy is associated with lower birth weight, babies that are small for their gestational age, and intrauterine growth restriction [18]. 

Vitamin D deficiency is accepted as an easily correctable contributing factor to intrauterine growth restriction (IUGR) and associated placental insufficiency. Although IUGR has frequently been attributed to placental insufficiency [19], maternal malnutrition may also play a role. Vitamin D plays an important physiological role in placental development, making lower serum levels of 25-(OH)D a threat to normal fetal growth and development. 

According to one study, women whose newborns showed signs of intrauterine growth restriction had, on average, 33% lower 25-(OH)D levels than women whose newborns showed normal intrauterine growth at the time of birth [20]. In this study, the mean 25-(OH)D level in pregnant women who gave birth to children with growth restriction was 16.8 mg/mL, compared with the 25.3 ng/mL median 25-(OH)D serum level in cases with normal growth. Emphasizing the importance of vitamin D sufficiency for normal fetal growth, a Cochrane review reported a greater than 50% reduction in low-birth-weight newborns in women who supplemented their vitamin D diet during pregnancy compared to newborns of women who did not receive vitamin D supplementation [21]. Furthermore, the risk for babies being small for their gestational age was 2.4 times higher if the mother’s 25-(OH)D serum level was less than 12 ng/mL, compared to 20 ng/mL or higher in the first trimester of pregnancy [21].

Recent studies have also shown that maternal vitamin D deficiency increases the risk of respiratory infections, wheezing, and other immune-related conditions in newborns [22].

Given the multiple correlations between vitamin D and maternal–fetal gestational pathology, it is important to thoroughly study the impact of vitamin D in pregnancy in order to prevent the onset of certain conditions.

## 2. Materials and Methods

This retrospective study was conducted at a tertiary maternity hospital in Bucharest, involving a cohort of 260 participants: 130 pregnant women and their 130 newborns, with vitamin D status measured in both groups. Over 95% of measurable serum vitamin D (25-OH) is vitamin D3 (25-OH), whereas vitamin D2 (25-OH) is only detectable in patients taking vitamin D2 supplements.

Vitamin D status was determined using the Elecsys Total Vitamin D Test, which employs a vitamin D binding protein (VDBP) as the capture protein. This protein binds to both vitamin D3 (25-OH) and vitamin D2 (25-OH). The Elecsys Total Vitamin D Test is designed for the quantitative measurement of total 25-hydroxyvitamin D in human serum and plasma, utilizing the immunochemical method of Electrochemiluminescence Immunoassay (ECLIA). Serum 25-hydroxyvitamin D (25-(OH)D) is the barometer for vitamin D status [23]. 

Serum levels of 25-(OH)D below 20 ng/mL (50 nmol/L) are generally thought to indicate moderate vitamin D deficiency, and below 10 ng/mL, severe deficiency. According to Mayo Medical Laboratories (Table 1), the total value of 25-hydroxyvitamin D2 and D3 is optimal between 20 and 50 ng/mL, while the range of 10–19 ng/mL shows a mild to moderate deficiency. A level lower than 10 ng/mL signals severe vitamin D deficiency [24].

Understanding the prevalence of vitamin D deficiency among pregnant women and newborns in the study population is essential. If the deficiency is relatively common, a sample size of 130 pregnant women should be adequate to detect significant differences.

Given the retrospective nature of our study, we applied specific inclusion and exclusion criteria during data collection to ensure the reliability and validity of the study population (Table 2 and Table 3).

In this study, after applying the inclusion and exclusion criteria, we identified 130 pregnant women and 130 newborns as reliable for analysis. 

Our study aimed to determine the effects of vitamin D deficiency during pregnancy, particularly on newborn outcomes. Considering that adequate vitamin D status is essential for the normal growth and development of the fetus, we aimed to establish a statistically significant relationship between maternal 25-(OH)D serum levels and newborn parameters such as birth weight, Apgar score, and their vitamin D status. Additionally, with vitamin D deficiency being associated with increased insulin resistance and a higher risk of anemia, we sought to examine the relationship between maternal vitamin D status and blood sugar/hemoglobin levels. It is well known that any impairment in maternal health during pregnancy can lead to complications for the newborn.

For statistical analysis, we used the SPSS program. We began the statistical analysis with a descriptive part, where we calculated the central tendency and dispersion parameters for the numerical variables. For the ordinal and nominal variables, we ran the frequency tables and extracted the main percentages.

Data representation was done using boxplots and line graphs. The Shapiro–Wilk test was applied to assess data distribution, revealing a non-normal distribution for most variables. Consequently, we used non-parametric tests such as the Mann–Whitney U test for comparisons between two groups, the Kruskal–Wallis test for comparisons among more than two groups, and the chi-squared test for proportions.

Furthermore, we conducted a risk analysis, calculating relative risk (RR) and odds ratio (OR) parameters for the sample and estimating the 95% confidence interval to determine if maternal vitamin D deficiency impacts newborn 25-(OH)D levels. Finally, we performed regression analysis to explore the connection between 25-hydroxivitamin D levels in mothers and their newborns. The confidence level for the entire study was set at α = 0.05.

## 3. Results

The database of 130 pregnant women was initially divided into two groups: those without vitamin D deficiency (35 subjects, 26.92%) serving as the control group, and those with vitamin D deficiency (95 subjects, 73.08%) constituting the study group. Based on the degrees of vitamin D deficiency, pregnant women were further categorized as follows, depending on 25-(OH)D serum levels: severe deficiency (under 10 ng/mL)—12 subjects (9.23%); moderate deficiency (between 10–20 ng/mL)—47 subjects (36.15%); mild deficiency (between 21–29 ng/mL)—36 subjects (27.7%); and optimal vitamin D status (between 30–100 ng/mL)—35 subjects (26.92%) (Table 4).

For the entire study, the subjects were analyzed according to these classifications. Vitamin D status (25-(OH)D serum levels) was also measured in the newborns, yielding the following distribution: severe deficiency (under 10 ng/mL)—5 subjects (3.85%); moderate deficiency (between 10–15 ng/mL)—19 subjects (14.62%); mild deficiency (between 16–19 ng/mL)—50 subjects (38.46%); and optimal vitamin D status (between 20–100 ng/mL)—56 subjects (43.07%). Most subjects with severe vitamin D deficiency (75%), and those with optimal status (74.28%), resided in urban areas. Gender did not influence vitamin D deficiency, as there was an almost equal distribution between genders.

**Figure 1:**
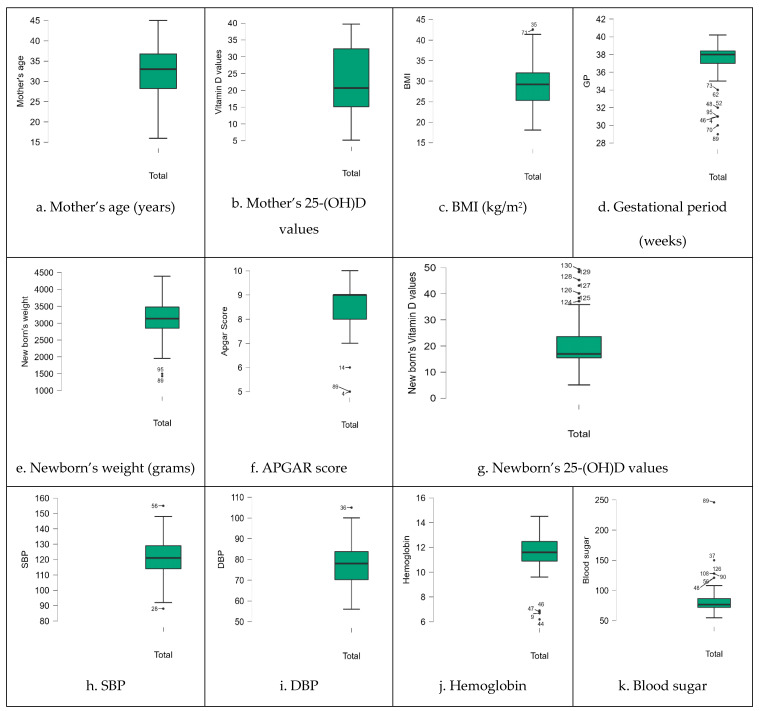
Boxplots of the data distribution for the whole sample.

For numerical variables, we considered the mother’s age, 25-(OH)D serum levels in both the mother and newborn, mother’s body mass index (BMI), gestational period (GP), newborn weight, APGAR score, systolic blood pressure (SBP), diastolic blood pressure (DBP), hemoglobin, and blood sugar. We calculated measures of central tendency and dispersion for the entire sample initially and subsequently after grouping the data according to the degrees of vitamin D deficiency. The complete analysis is presented in the following tables and figures (Figure 1, Table 4 and Table 5).

**Figure 2:**
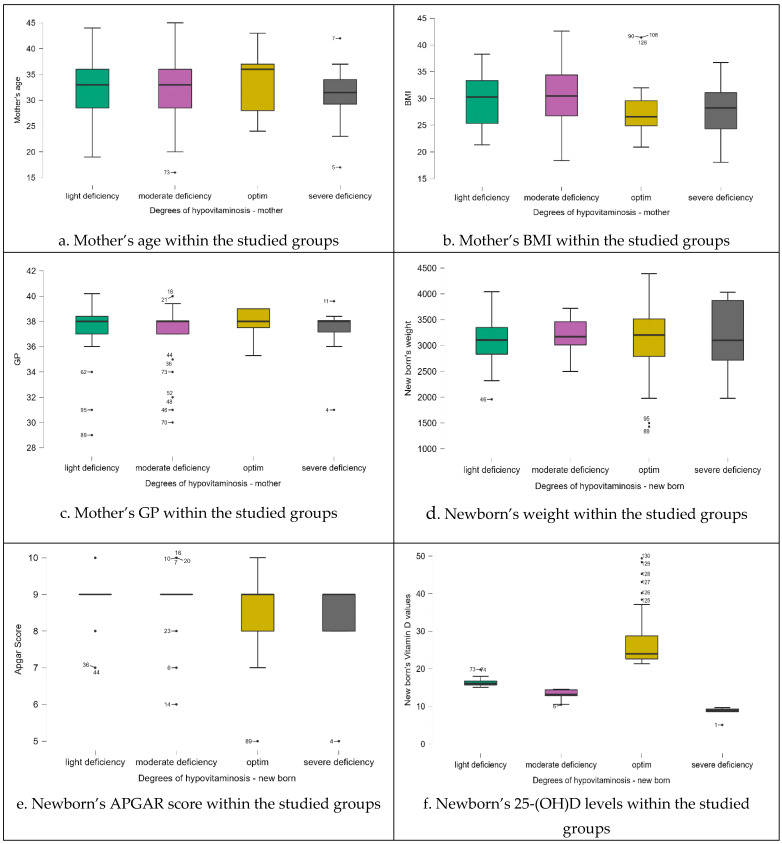
Boxplots of the data distribution for the subjects split according to their vitamin D status.

**Figure 3:**
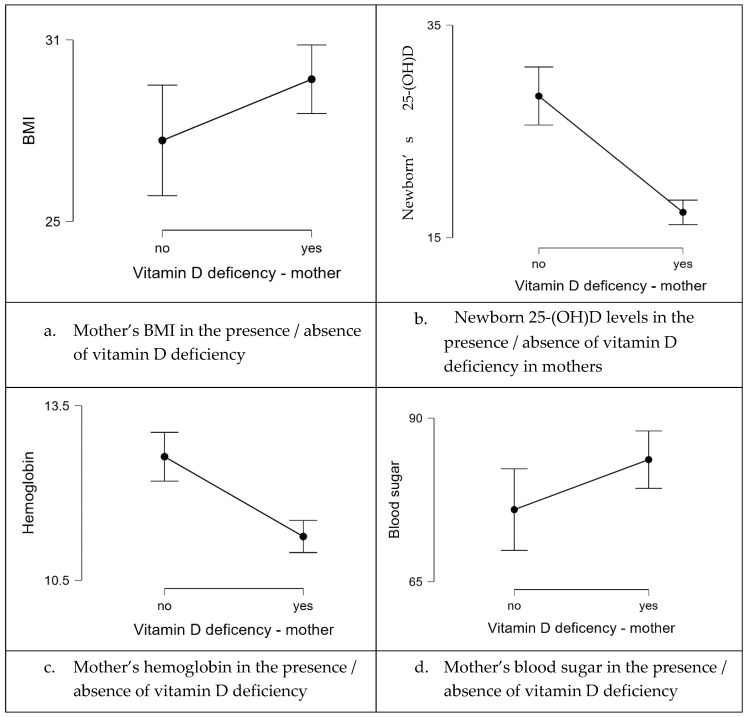
Graphical representation of the significant results obtained through the above analysis.

In the case of our group, according to Table 5, BMI is influenced by the deficiency of vitamin D (Table 5, Figure 2 and Figure 3).

We ran descriptive statistics for the numerical variables of the study, and we split them based on the degrees of vitamin D deficiency in newborns (Table 6).

Because there are differences between the tested groups, several statistical tests were applied to test the data’s significance. In the beginning, the Mann–Whitney test was used to see if there were any significant differences between the numerically studied variables in the presence or absence of vitamin D deficiency (Table 7). Significant results (*p* < 0.05) were obtained in the cases of BMI, APGAR score, the newborn’s 25-(OH)D mean values, and the mother’s hemoglobin and blood sugar mean values. Overall, the patients with normal 25-(OH)D values were found to be healthier.

**Figure 4:**
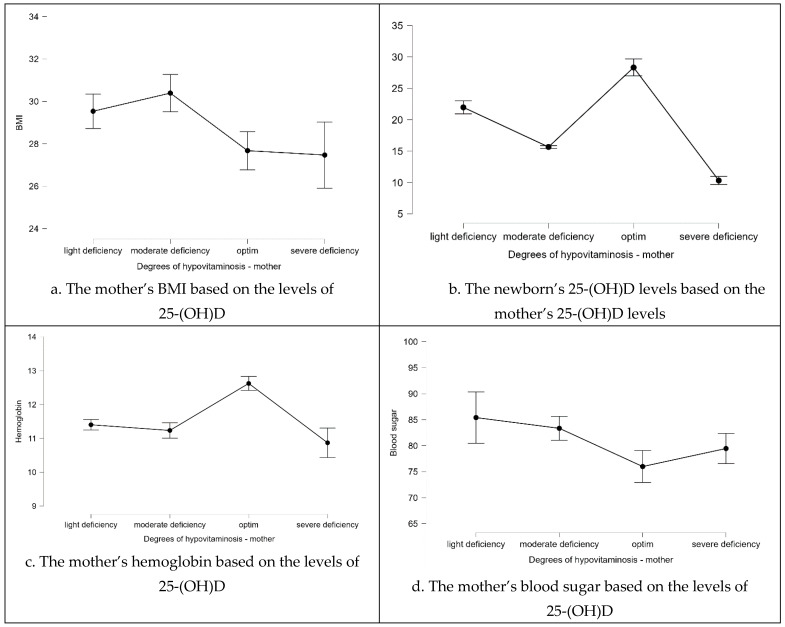
Graphical representation obtained from the Kruskal–Wallis test.

Based on the obtained results, we tested the significant results regarding the degrees of vitamin D deficiency using the Kruskal—Wallis test (Figure 4). So, for BMI, we again obtained significant results (*p* = 0.035, statistics = 7.7). The lowest BMI was seen in patients with severe vitamin D deficiency. One of the most important results obtained up to this point was that the children of the patients with severe vitamin D deficiency have as well severe vitamin D deficiency in most of the cases (*p* < 0.001, statistics = 96.686). In addition, as in the previous test, in the case of the Kruskal—Wallis test, we obtained significant results in the cases of hemoglobin (*p* < 0.001, statistic = 27.821) and blood sugar (*p* = 0.002, statistics = 15.126). As can be seen in Figure 4, women with a severe deficit of vitamin D also have severe anemia as well as high blood sugar values.

The main part of this analysis is testing to see if we can consider the mother’s deficiency in vitamin D as a risk factor for the newborn, so for that we split the data into a contingency table (Table 8), obtaining an extremely significant risk factor (*p* < 0.001, *RR* > 1, *OR* > 1), so we can conclude that if the mother has a vitamin D deficiency, it increases the chance that the newborn will have a vitamin D deficiency more than eight times. The chi-squared test was used in this case.

Having vague clinical studies on markers that can predict the risk of thrombosis during pregnancy and changes in coagulation factors in pregnant women with thrombophilia or pregnant women at risk of preeclampsia, we also discussed BP values [25,26,27].

Seeing these results, we tested to see if these degrees of vitamin D deficiency can increase the risk for the mother to develop other diseases, and we obtained significant results (*p* < 0.05) in the cases of: high blood pressure induced by pregnancy (*p* = 0.048, statistics = 7.75); autoimmune thrombophilia (*p* = 0.037, statistics = 6.905); preeclampsia (*p* = 0.013, statistics = 10.766); and anemia (*p* = 0.027, statistics = 14.787). So, we can say that vitamin D deficiency can cause several dangerous diseases during pregnancy. 

**Figure 5:**
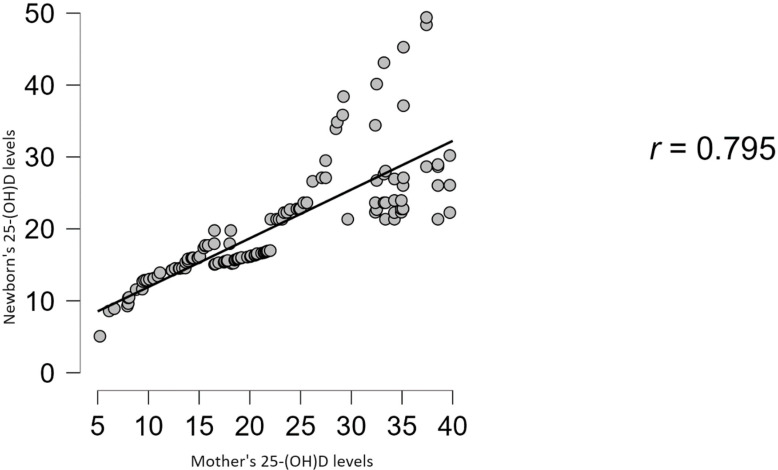
The association between the mother’s and the newborn’s vitamin D status using a scatter plot graph.

At the end of our study, we ran a linear regression model (Figure 5) to see if we could find an association between the mother’s and newborn’s vitamin D status, obtaining a significant, strong, positive, and direct correlation (*r* = 0.795, R^2^ = 0.632, *p* < 0.001) meaning that these two medical tests are extremely dependent on one another. The relationship between these variables is plotted using a scatter plot graph.

## 4. Discussion

Worldwide, vitamin D deficiency during pregnancy and the post-partum period is a significant public health concern [28,29,30]. When investigating vitamin D deficiency during pregnancy, it is important to take into consideration, in addition to vitamin D supplementation, lifestyle factors as well. Studies have shown that there are many lifestyle factors associated with vitamin D deficiency, such as limited sun exposure due to geographical location, indoor lifestyle, clothing choices, use of sunscreen that can significantly reduce the skin’s ability to produce vitamin D, dietary habits, skin pigmentation, and certain medical conditions that can affect the body’s ability to absorb or metabolize vitamin D effectively [31]. 

Numerous studies have investigated the impact of vitamin D deficiency on placental function and inflammatory responses during pregnancy. As for placental inflammation, vitamin D has been recognized as having an essential role in its suppression. There is substantial evidence linking vitamin D deficiency to gestational diabetes, preterm birth, spontaneous abortion, preeclampsia, and other complications [15,32,33,34,35,36,37,38,39,40,41,42]. Our study initially measured maternal vitamin D status, recognizing that a deficiency in serum 25-(OH) D can adversely affect the fetus.

Several studies have demonstrated an inverse relationship between maternal 25-(OH)D serum levels and increased adiposity in children and adolescents [41,42,43]. The pathophysiology of increased adiposity and its direct relationship with vitamin D deficiency can be explained by the decreased bioavailability of vitamin D stored in adipose tissue, which reduces its metabolic activation and consumption by the maternal body [44]. Some research indicates that maternal obesity is associated with higher birth weights and increased fetal adiposity, both of which suggest a higher risk of obesity in offspring [45,46,47,48]. Maternal obesity combined with vitamin D deficiency, whether in the mother or fetus, should not be misdiagnosed as gestational diabetes, as some studies suggest [49,50,51,52]. Nevertheless, the frequent co-occurrence of obesity and vitamin D deficiency should not be overlooked [53,54].

Vitamin D deficiency in newborns has been implicated in various neonatal disorders. Clinical studies indicate that placental changes influence the action of vitamin D, underscoring the importance of adequate vitamin D intake during pregnancy [55,56,57]. To prevent the consequences of vitamin D deficiency in newborns, it is recommended that pregnant women be tested in the first trimester to avoid hypocalcemia secondary to vitamin D deficiency, which can lead to seizures in newborns [58]. Maintaining adequate vitamin D status during pregnancy is crucial for the healthy development of the fetus, taking into consideration that deficiency has been associated with intrauterine growth restriction, babies that are small for their gestational age, and a lower birth weight [22]. Furthermore, vitamin D status influences the development and function of the immune system. Deficiency may impair the fetal immune response, potentially leading to increased susceptibility to infections and a higher risk of developing autoimmune diseases later in life. Also, vitamin D deficiency may be linked to an increased risk of respiratory issues in newborns, such as wheezing and respiratory infections [22].

Given the numerous correlations between vitamin D and maternal–fetal gestational pathology, it is crucial to study the impact of vitamin D during pregnancy to prevent certain diseases, thereby reducing mortality and morbidity. This necessitates adjusting optimal doses of vitamin D in new clinical protocols, thereby contributing to the continuous improvement of our medical system [59].

## 5. Conclusions

It is well known that pregnancy involves a dynamic period of physiological changes for both the mother and the fetus. Numerous observational studies have demonstrated the crucial role of vitamin D during pregnancy, particularly in cases where pregnant women are supplemented with the necessary vitamin D to maintain maternal and fetal well-being, as observed in our group. Despite this, vitamin D deficiency remains prevalent. Our findings indicate that vitamin D supplementation during pregnancy contributed to full-term births rather than preterm deliveries. However, the high number of mothers with vitamin D deficiency in our group suggests that supplementation was insufficient, and that the dietary intake of pregnant women was inadequate. Therefore, it is essential to investigate vitamin D status in the first trimester and provide individualized supplementation based on each specific case.

Further research is necessary to determine the effects of vitamin D deficiency on the mother and the newborn and to establish the link between this deficiency and various serious diseases that the mother may develop during pregnancy.

## Figures and Tables

**Table 1:** Vitamin D status.

Serum Level of 25-hydroxyvitamin D	Vitamin D Status
20–50 ng/mL	Optimal
10–19 ng/mL	Mild to moderate deficiency
<10 ng/mL	Severe deficiency

**Table 2:** Inclusion and exclusion criteria regarding pregnant women.

Inclusion Criteria	Exclusion Criteria
Age range—pregnant women aged 16–45 years to ensure the study population is within the typical reproductive age	Pre-existing conditions—chronic kidney disease, malabsorption syndromes, or autoimmune diseases that could independently affect vitamin D status or pregnancy outcomes
Gestational age—the gestational age at which the birth took place was between 37 and 40 weeks	Supplementation variability
Residence—pregnant women who lived in the same study area to avoid regional variation in vitamin D exposure	Drug use—anticonvulsants or corticosteroids that are known to interfere with vitamin D metabolism
Singleton pregnancy—in order to avoid complications related to multiple pregnancies	Multiple pregnancies
Consistent medical records—including vitamin D status, demographic information, and obstetric history	Inconsistent medical records

**Table 3:** Inclusion and exclusion criteria regarding newborns.

Inclusion Criteria	Exclusion Criteria
Live births	Congenital anomalies
Complete medical records—including birth weight, gestational age at birth, Apgar score, and vitamin D status	Incomplete records
	Preterm births—prematurity could confound the effects of vitamin D deficiency

**Table 4:** Descriptive statistics run for the numerical variables of the study.

Statistical Analysis	Mother’s Age	25-(OH)Dvalues	BMI	GP	Newborn Weight	Apgar Score	Newborn 25-(OH)D Values	SBP	DBP	Hemoglobin	Blood Sugar
Valid data	130	130	130	130	130	130	130	130	130	130	130
Mode	36	34.25	20.9	38	2980	9	21.34	117	70	11.6	72
Median	33	20.69	29.22	38	3135	9	16.95	121	78	11.6	76.61
Mean	32.42	22.43	29.16	38	3107.3	9	20.33	121.7	78.5	11.6	81.58
Standard deviation	6.02	9.38	5.55	1.94	544.2	0.9	7.98	11.4	9.57	1.45	20.88
Standard error of the mean	0.53	0.82	0.49	0.17	47.73	0.08	0.7	1	0.84	0.13	1.83
*p*-value from Shapiro—Wilk	0.015	<0.001	0.08	<0.001	0.06	<0.001	<0.001	0.01	0.04	<0.001	<0.001
Range	29	34.49	24.53	11.2	296	5	44.32	67	49	8.3	191
Minimum	16	5.2	18.07	29	143	5	5.07	88	56	6.2	55
Maximum	45	39.71	42.6	40.2	439	10	49.39	155	105	14.5	246

**Table 5:** Descriptive statistics ran for the numerical variables of the study, split based on the degrees of vitamin D deficiency.

Statistical Analysis	Mother’s Age (Years)				BMI (kg/m^2^)				GP (Weeks)			
	Severe Deficiency	Moderate Deficiency	Light Deficiency	Optimal Vitamin D Status	Severe Deficiency	Moderate Deficiency	Light Deficiency	Optimal Vitamin D Status	Severe Deficiency	Moderate Deficiency	Light Deficiency	Optimal Vitamin D Status
Valid data	12	47	36	35	12	47	36	35	12	47	36	35
Mode	31	33	26	36	18.07	30.48	21.33	20.9	38	38	38	39
Median	31.5	33	33	36	28.21	30.48	30.23	26.57	38	38	38	38
Mean	31.08	31.92	32.22	33.77	27.47	30.39	29.54	27.68	37.29	31.31	37.49	37.95
Standard deviation	6.59	6.15	5.89	5.81	5.42	6.02	4.89	5.32	2.16	2.18	2.2	1.07
Standard error of the mean	1.9	0.89	0.98	0.98	1.57	0.88	0.82	0.89	0.62	0.32	0.37	0.18
*p*-value from Shapiro—Wilk	0.7	0.647	0.42	<0.001	0.86	0.69	0.265	<0.001	<0.001	<0.001	<0.001	<0.001
Range	25	29	25	19	18.65	24.23	16.95	20.5	8.6	10	11.2	3.7
Minimum	17	16	19	24	18.07	18.37	21.33	20.9	31	30	29	35.3
Maximum	42	45	44	43	36.72	42.6	38.28	41.4	39.6	40	40.2	39

**Table 6:** Descriptive statistics for the numerical variables of the study, split based on the degrees of vitamin D deficiency in newborns.

Statistical Analysis	Newborn’s Weight (Grams)				APGAR Score				Newborn’s 25-(OH)D Values (ng/mL)			
	Severe Deficiency	Moderate Deficiency	Light Deficiency	Optimal Vitamin D Status	Severe Deficiency	Moderate Deficiency	Light Deficiency	Optimal Vitamin D Status	Severe Deficiency	Moderate Deficiency	Light Deficiency	Optimal Vitamin D Status
Valid data	5	19	50	56	5	19	50	56	5	19	50	56
Mode	1980	3100	3250	2990	9	9	9	9	5.07	12.87	15.97	21.34
Median	3100	3170	3105	3202.5	9	9	9	9	8.91	13.12	16.05	23.95
Mean	3140	3193.42	3070.9	3107.77	8	8.89	8.89	8.63	8.29	13.17	16.34	27.4
Standard deviation	843.89	320.79	440.16	657.79	1.73	0.99	0.69	0.93	1.84	1.59	1.03	7.17
Standard error of the mean	377.39	73.59	62.25	87.9	0.78	0.23	0.09	0.13	0.83	1.32	0.15	0.96
*p*-value from Shapiro—Wilk	0.69	0.88	0.59	0.036	0.01	<0.001	<0.001	<0.001	0.029	0.018	<0.001	<0.001
Range	2050	1220	2080	2960	4	4	3	5	4.55	4.16	4.71	28.05
Minimum	1980	2500	1960	1430	5	6	7	5	5.07	10.4	15.06	21.34
Maximum	4030	3720	4040	4390	9	10	10	10	9.62	14.56	19.77	49.39

**Table 7:** The analysis given by the Mann–Whitney U test. The significant results (*p* < 0.05) were highlighted in bold.

Variables	W	P
Mother’s age	1997.500	0.078
BMI	1212.000	**0.018**
GP	1939.000	0.138
Newborn’s weight	1587.000	0.694
APGAR Score	1164.500	**0.003**
Newborn’s Vitamin D status	3041.500	**<0.001**
SBP	1392.500	0.157
DBP	1434.500	0.232
Hemoglobin	2651.000	**<0.001**
Blood sugar	932.000	**<0.001**

**Table 8:** Correlation between maternal and newborn vitamin D deficiency.

Contingency Table			Results
Variables	Newborn with vitamin D deficiency	Newborns without vitamin D deficiency	*p* < 0.001 *RR* = 8.84, 95% CI ∈ (2.98; 26.25) *OR* = 33.39, 95% CI ∈ (9.35;119.28)
Mothers with vitamin D deficiency	72	23	
Mothers without vitamin D deficiency	3	32	

## Data Availability

The data presented in this study are available on reasonable request form the corresponding author.
